# Summary report of the Standards, Options and Recommendations for the use of serum tumour markers in breast cancer: 2000

**DOI:** 10.1038/sj.bjc.6601082

**Published:** 2003-08-15

**Authors:** J P Basuyau, M P Blanc-Vincent, J M Bidart, A Daver, L Deneux, N Eche, G Gory-Delabaere, M F Pichon, J M Riedinger

**Affiliations:** 1 Centre Henri Becquerel, Rouen, France; 2 FNCLCC, Paris, France; 3 Institut Gustave Roussy, Villejuif, France; 4 Centre Paul Papin, Angers, France; 5 Institut Curie, Paris, France; 6 Institut Claudius Regaud, Toulouse, France; 7 Centre René Huguenin, Saint-Cloud, France; 8 Centre Georges-François Leclerc, Dijon, France

**Keywords:** breast neoplasms, tumour markers, practice guideline

## 

Although several clinical practice guidelines have been produced for the use of serum tumour markers in the management of breast cancer, the practice remains inconsistent (ASCO, 1996; ANAES, 1997; Bast *et al*, 2001; Mauriac *et al*, 2001). All current knowledge was therefore reviewed.

## OBJECTIVES

The objective was to define the characteristics of breast cancer tumour markers and identify their potential role in the management of patient with cancer. The main question concerns the role of CA 15-3 (and to a lesser degree that of carcinoembryonic antigen (CEA)) in the early diagnosis of metastases and locoregional recurrence and in evaluation of response to treatment.

These recommendations are intended for both scientists and clinicians. This document does not cover the clinical indications in which tumour markers should or can be used. This aspect is covered in the Standards, Options and Recommendations (SORs) document on metastatic breast cancer (Mauriac *et al*, 2001). This document aims to provide the multidisciplinary team, responsible for defining the treatment plan, with laboratory data to help them optimise the management of patients with metastatic breast cancer.

## METHODOLOGY FOR THE DEVELOPMENT OF THE SORS FOR SERUM TUMOUR MARKERS IN BREAST CANCER

The general methodology has been previously described (Fervers *et al*, 2001). For this particular SOR, a working group of scientists and clinicians was set up by the National Federation of French Cancer Centres (Fédération Nationale des Centres de Lutte Contre le Cancer: FNCLCC) to review the available scientific data for the use of serum tumour markers in breast cancer. A literature search of *Medline®*, from 1991 to 1998, was updated in 2000. The members of the working group also provided references from their personal reference database. The members of the group selected and critically appraised the papers and then wrote a draft version of the clinical practice guidelines, the ‘Standards’, ‘Options’ and Recommendations' (Fervers *et al*, 2001) for breast cancer, based on the available scientific evidence or expert agreement.

‘*Standards*’ identify clinical situations for which there exist strong indications or contraindications for a particular intervention and ‘*Options*’ identify situations for which there are several alternatives, none of which have shown clear superiority over the others (Table 1

**Table 1:** Definition of ‘Standards, Options and Recommendations’

*Standards*	Procedures or treatments that are considered to be of benefit, inappropriate or harmful by unanimous decision based on the best available evidence
	
*Options*	Procedures or treatments that are considered of to be benefit, inappropriate or harmful by a majority, based on the best available evidence
	
*Recommendations*	Additional information to enable the available options to be ranked using explicit criteria (e.g., survival, toxicity) with an indication of the level of evidence

). In any SOR, there can be several ‘*Options*’ for a given clinical situation. ‘*Recommendations*’ enable the ‘*Options*’ to be weighted according to the available evidence. Several interventions can be recommended for the same clinical situation, so that clinicians can make a choice according to specific clinical parameters, for example, local circumstances, skills, equipment, resources and patient preferences. Adapting the SORs to a local situation is possible if the reason for the choice is sufficiently transparent and this is crucial for successful implementation. Inclusion of patients in clinical trials is an appropriate form of patient management in oncology and is recommended frequently within the SORs, particularly in situations where the evidence is too weak to support an intervention.

The type of evidence underlying any ‘*Standard*’, ‘*Option*’ or ‘*Recommendation*’ is indicated using a classification developed by the FNCLCC based on previously published models. The level of evidence depends not only on the type and quality of the studies reviewed, but also on the concordance of the results (Table 2

**Table 2:** Definition of level of evidence

*Level A*
There exists a high standard meta-analysis on several high standard randomized clinical trials that give consistent results

*Level B*
There exists good quality evidence from randomised trials (B1) or prospective or restrospective studies (B2). The results are consistent when considered together

*Level C*
The methodology of the available studies is weak or their results are not consistent when considered together

*Level D*
Either the scientific data does not exist or there is only a series of cases

*Expert agreement*
The data does not exist for the method concerned, but the experts are unanimous in their judgement

). When no clear scientific evidence exists, judgement is made according to the professional experience and consensus of the expert group (‘expert agreement’).

The full document was then reviewed by independent experts and the final document was validated in April 2000. This summary version was drafted in January 2001 from the full version (Basuyau *et al*, 2000) and both versions are available in French on the FNCLCC web site: http://www.fnclcc.fr. The SORs will be updated when new scientific data becomes available or if there is a change in expert agreement.

## SUMMARY OF SORS FOR SERUM TUMOUR MARKERS IN BREAST CANCER

CA 15.3 and to a lesser extent CEA are the most commonly used serum tumour markers in breast cancer. If the concentration of CA 15.3 is high at presentation, routine assays of other markers is not justified (standard, expert agreement). All assays for a given patient should be performed in the same laboratory, using the same technique, since results have been shown to be dependant on the assay technique used (standard, expert agreement). Other markers, mainly epitopes present on mucin glycoproteins (CA549, CA M26, CA M29, CA27.29) are under evaluation, but to date none have been shown to be more useful than CA 15.3. These other markers may be used instead of CA 15.3, but not in combination.

### Role of CA 15.3 assays in the screening and diagnosis of breast cancer

The assay for CA 15.3 should not be used as a screening or diagnostic test because of its low sensitivity (standard, level of evidence: B2). In the diagnosis of metastatic adenocarcinoma of unknown origin, CA 15.3 is likely to help make the diagnosis and therefore will have an impact on the treatment plan (option, expert agreement).

### Significance of the initial level of CA 15-3

The pretreatment CA 15.3 level is a recognised prognostic factor, but it has not been proven to be an independent prognostic factor (standard, level of evidence: B2).

An initially high CA 15.3 level is more often observed in patients with advanced cancer than in those with localised cancer (standard, level of evidence: B2). Several studies have shown that the concentration is correlated with the disease stage (standard, level of evidence: B2). If the initial concentration is more than 50 kU l^−1^, a search for metastases should be undertaken before any treatment plan is decided (standard, expert agreement), particularly if the result of this search will modify treatment.

The pretreatment concentration of CA 15.3 should be considered as the reference value, in the event of suspected metastatic recurrence (recommendation, expert agreement).

### Prognostic value of a rising CA 15.3 level during the initial treatment

CA 15.3 levels that are initially high and remain high despite treatment indicate a failure to respond to treatment and a very poor prognosis (standard, level of evidence: C).

### Early diagnosis of locoregional and/or metastatic recurrence

The sensitivity of tumour markers in the diagnosis of local recurrence is poor, but their usefulness (particularly that of CA 15.3) in the early diagnosis of breast cancer metastases is clear (standard, level of evidence: A).

The early detection of metastatic disease does not benefit the patient in terms of overall survival or time to the appearance of clinical signs (standard, level of evidence: C).

### Role of tumour markers in the follow-up after treatment

The CA 15.3 level at the time of the diagnosis of metastatic disease does not seem to be a prognostic factor for treatment response (standard, level of evidence: C). There is a correlation between tumour marker levels and disease response during treatment for metastases (standard, level of evidence: C). The measurement of CA 15.3 levels during treatment follow-up in patients with metastatic disease is useful in the evaluation of the treatment response, but should not replace clinical examination (standard). Prospective clinical trials using the criteria defined by the Working Group on Tumor Marker Criteria (Bonfrer, 1990) and evaluating the rate of change in tumour marker levels should be undertaken (recommendation, expert agreement).

### Combination of different tumour markers

There is no justification for routinely measuring a number of markers (standard, level of evidence: B2). CA 15.3 remains the reference marker for breast cancer (standard). Other markers should only be measured in the setting of a randomised clinical trial assessing the benefits of the early diagnosis and treatment of metastases (recommendation, expert agreement).

If the concentration of CA 15.3 remains normal, but there is obvious clinical progression of disease, alternative indicators for treatment response should be identified such as CEA, tissue polypeptide antigen (TPA), or polypeptide-specific antigen (TPS) (recommendation, expert agreement).

## Appendix

### Reviewers

F Bonnet (Institut Claudius Regaud, Toulouse), J Bonneterre (Centre Oscar Lambret, Lille), A Chevrier (Centre Henri-Becquerel, Rouen), B De La Lande (Centre René Huguenin, Saint-Cloud), L Demange (Institut Jean Godinot, Reims), M Durand (Institut Bergonié, Bordeaux), JM Dilhuydy (Institut Bergonié, Bordeaux), JR Garbay (Institut Gustave Roussy, Villejuif), P Kerbrat (Centre Eugène Marquis, Rennes), A Lortholary (Centre Paul Papin, Angers), M Marty (Institut Gustave Roussy, Villejuif), L Mauriac (Institut Bergonié, Bordeaux), A Pecking (Centre René Huguenin, Saint-Cloud), H Pujol (Ligue nationale contre le cancer, Paris), B Querleu (CHU Pavillon Paul Gellé, Roubaix), and M Spielmann (Institut Gustave Roussy, Villejuif).
